# Using Probiotics to Flatten the Curve of Coronavirus Disease COVID-2019 Pandemic

**DOI:** 10.3389/fpubh.2020.00186

**Published:** 2020-05-08

**Authors:** David Baud, Varvara Dimopoulou Agri, Glenn R. Gibson, Gregor Reid, Eric Giannoni

**Affiliations:** ^1^Materno-Fetal and Obstetrics Research Unit, Department Woman-Mother-Child, Lausanne University Hospital, University of Lausanne, Lausanne, Switzerland; ^2^Clinic of Neonatology, Department Woman-Mother-Child, Lausanne University Hospital, University of Lausanne, Lausanne, Switzerland; ^3^Food and Nutritional Sciences, St Joseph's Hospital, The University of Reading, Reading, United Kingdom; ^4^Department of Microbiology and Immunology, The University of Western Ontario, London, ON, Canada; ^5^Canadian R&D Centre for Human Microbiome and Probiotics, Lawson Health Research Institute, London, ON, Canada

**Keywords:** COVID 19, probiotics, prebiotics, SARS-CoV-2, pandemics, coronavirus, respiratory infection

## Introduction

Despite strategies based on social distancing, hygiene, and screening, COVID-19 is progressing rapidly throughout the world, with healthcare systems at risk of being overwhelmed. While identification of effective drug therapies is ongoing, vaccines will not be available in the near future. Therefore, additional preventive strategies are urgently needed.

COVID-19 presents with a spectrum of disease severity, ranging from mild and non-specific flu-like symptoms, to pneumonia, and life-threatening complications such as acute respiratory distress syndrome (ARDS) and multiple organ failure. While transmission of SARS-CoV-2 is thought to occur mainly via respiratory droplets, the gut may also contribute toward the pathogenesis of COVID-19 (1). SARS-CoV-2 RNA has been detected in the gastrointestinal tract and stool samples from patients (2–4), and in sewage systems (5). Coronaviruses, including SARS-Cov-2 can invade enterocytes, thereby acting as a reservoir for the virus (4). Indeed, large clinical studies from China indicate that gastrointestinal symptoms are common in COVID-19, and are associated with disease severity (3, 4).

Probiotics are live microorganisms that when administered in adequate amounts confer a health benefit on the host (6). Clinical evidence shows that certain probiotic strains help to prevent bacterial and viral infections, including gastroenteritis, sepsis, and respiratory tract infections (RTIs). The reason for adding probiotic strains to the overall prevention and care strategy is founded in science and clinical studies, albeit hitherto none directly on the etiological agent of this pandemic.

## Clinical Data Supporting the Use of Probiotics to Prevent Covid-19

Probiotics can prevent antibiotic-associated diarrhea, and infections in the gastrointestinal tract, but also infections at other sites, including sepsis, and RTIs (7–13). Meta-analyses are the gold standard for evidence-based medicine. In one analysis of more than 8,000 preterm infants included in randomized control trials (RCTs), patients receiving enteral supplementation with probiotics showed a reduction in necrotizing enterocolitis, nosocomial sepsis, and all-cause mortality (14). A well-conducted RCT including >4,000 newborns in India found a reduction in sepsis and lower RTIs in infants treated with a strain of *Lactobacillus plantarum* combined with prebiotics (which are growth substrates specific for beneficial microorganisms) (15).

Viruses are etiologic agents of over 90% of upper RTIs. The positive impact of probiotics on prevention of upper RTIs is documented in a number of studies. A meta-analysis of 12 RCTs including 3,720 adults and children reported a 2-fold lower risk of developing upper RTI in subjects taking probiotics, and a small but significant reduction in disease severity in those infected. A randomized, double-blind, placebo-controlled intervention study of 479 adults showed that *Lactobacillus gasseri* PA 16/8, *Bifidobacterium longum* SP 07/3, and *Bifidobacterium bifidum* MF 20/5 with vitamins and minerals lowered not only the duration of common cold episodes but also days with fever (16). The impact of probiotics on prevention of upper RTIs caused by specific viruses has also been documented. An RCT including 94 preterm infants showed that galacto-oligosaccharide and polydextrose prebiotic mixture (1:1), or probiotic *Lactobacillus rhamnosus* GG given between 3 and 60 days of life lowered the incidence of clinically defined virus-associated RTI by 2- to 3-fold compared to placebo (17). The incidence of rhinovirus-associated episodes, which comprised 80% of all RTIs in this study, was also strongly reduced with probiotics or prebiotics. The incidence of influenza RTI was reduced following consumption of *Lactobacillus brevis* in an open label study of 1,783 school children (18). Pertinent to the pandemic affecting adults more than children, these positive findings were confirmed in an RCT that included 27 elderly subjects receiving *Bifidobacterium longum* or placebo (19). Furthermore, lactic acid bacteria, from which many probiotics are selected, are part of the upper respiratory tract microbiota in healthy people, and some strains are being considered for prevention of recurrent otitis media (20, 21). This makes their use for contributing to slow down progression of the coronavirus pandemic worthy of consideration.

Probiotics have also been used to prevent bacterial lower RTIs in critically ill adults. Meta-analyses of RCTs including close to 2,000 patients found that probiotic strains reduce the incidence of ventilator-associated pneumonia (22, 23). But low quality of evidence and conflicting results among different studies calls for additional well-conducted RCTs.

It should be noted that not all probiotics, even those with gastrointestinal benefits, necessarily contribute in every way to reducing the risk of respiratory infection. For example, *Lactobacillus rhamnosus* GG and *Bifidobacterium animalis* ssp. *lactis* may contribute to intestinal benefits, but do not reduce the number of viruses in the nasopharynx (24). Examples of products that could be considered, depending on availability in a given country, are provided in Table 1.

**Table 1:** The following are examples (not exclusive) of probiotic products, or web sites listing products, with documentation in human studies that may have relevance to reducing the burden of the coronavirus pandemic.

**Products**	**Basis for inclusion**	**When to administer**	**References**
*Lactobacillus casei* DN-114 001; DanActive/Actimel Fermented drink, Danone	Reduced incidence and duration of RTIs	Once daily for duration of the pandemic	(12, 13)
*Lactobacillus gasseri* PA 16/8, *Bifidobacterium longum* SP 07/3, and *B. bifidum* MF 20/5; Tribion harmonis, Merck	Lowering duration and severity of flu-like illness	Once daily for duration of the pandemic	(16)
*Lactobacillus rhamnosus* GG; Culturelle or other brand names	For digestive health and gut barrier integrity, and prevention of viral RTIs	One capsule daily for duration of the pandemic	(17)
*Lactobacillus plantarum* DR7; Malaysia	Prevention of upper RTIs, immune modulation	2 g sachet per day for duration of pandemic	(25)
*Bifidobacterium breve* Yakult, and *Lactobacillus casei* Shirota; available as fermented drinks	Lower incidence of ventilator-associated pneumonia	One of each day for duration of the pandemic	(26)
*Bifidobacterium longum* BB536; Morinaga, and sold in many formulations	Enhances innate immunity, prevents influenza infection	One each day for duration of the pandemic	(19)
*Pediococcus pentosaceus* 5-33:3, *Leuconostoc mesenteroides* 32-77:1, *L. paracasei* ssp. *paracasei* 19, *L. plantarum* 2,362 plus inulin, oat bran, pectin, and resistant starch; Medipharm, Sweden	To reduce rate of SIRS, infections, sepsis, days of stay in the intensive care unit, days under mechanical ventilation, and mortality	For COVID-19 patients	(27)
A list of probiotics available in Canada for various health issues; www.probioticchart.ca			
A list of probiotics available in the USA for various health issues; www.usprobioticguide.com			

*We must emphasize that none have been tested or proven to have an effect against SARS-CoV2, the virus causing COVID-19, nor are they proven treatments or cures for this condition*.

## Mechanistic Basis for the Action of Probiotics to Prevent Infections and Relevance to Covid-19

Mechanisms that might explain clinical success of probiotics include enhancement of the intestinal epithelial barrier, competition with pathogens for nutrients and adhesion to the intestinal epithelium, production of anti-microbial substances and modulation of the host immune system (28). An RCT of 55 infants showed that enteral supplementation with a combination of *Bifidobacterium bifidum* and *Streptococcus thermophilus* reduced the incidence of diarrhea and shedding of rotaviruses (29), an effect that has been confirmed in subsequent studies (30). This would indicate interference with viral entry into cells and/or inhibition of viral replication in the intestine. While this mechanism may have a role in reducing dissemination of coronavirus via the gut, the probiotic strains were not administered to the respiratory tract. So, direct inhibition may appear impossible at this site. Having said that, lungs have their own microbiota and a gut-lung connection has been described whereby host-microbe, microbe-microbe and immune interactions can influence the course of respiratory diseases (31). RTIs such as influenza are associated with an imbalance in the microbial communities of the respiratory and gastrointestinal tracts (32, 33). This dysbiosis may alter subsequent immune function and predispose to secondary bacterial infection. As reports from China indicate that COVID-19 might be associated with intestinal dysbiosis causing inflammation and poorer response to pathogens (34, 35), the case exists for probiotic strains that restore gut homeostasis (36). It is feasible that orally administered probiotic strains could further influence this gut-lung axis, as some can migrate from the gut to distant sites, such as the breast to treat mastitis (37).

The gut microbiome has a critical impact on systemic immune responses, and immune responses at distant mucosal sites, including the lungs (38, 39). Administration of certain bifidobacteria or lactobacilli has beneficial impact on influenza virus clearance from the respiratory tract (39, 40). Probiotic strains improve levels of type I interferons, increase the number and activity of antigen presenting cells, NK cells, T cells, as well as the levels of systemic and mucosal specific antibodies in the lungs (16, 19, 39). There is also evidence that probiotic strains modify the dynamic balance between proinflammatory and immunoregulatory cytokines that allow viral clearance while minimizing immune response-mediated damage to the lungs. This might be particularly relevant to prevent ARDS, a major complication of COVID-19. An RCT with *Lactobacillus plantarum* DR7 showed suppression of plasma pro-inflammatory cytokines (IFN-γ, TNF-α) in middle-aged adults, and enhancement of anti-inflammatory cytokines (IL-4, IL-10) in young adults, along with reduced plasma peroxidation and oxidative stress levels (25). Given the cytokine storm that appears to occur in many COVID-19 patients, this type of modulation may prove to be very important. The manner in which orally administered probiotic strains contributes to this appears to involve the immune response emanating from the intestine, a focal point of the body's defenses. Therefore, probiotic strains documented to enhance the integrity of tight junctions, for example through increasing butyrate, a fuel for colonocytes could theoretically reduce SARS-Cov-2 invasion.

Evidence for antiviral activity of probiotic strains against common respiratory viruses, including influenza, rhinovirus, and respiratory syncytial virus comes from clinical and experimental studies (17–19, 41). While none of these effects or mechanisms have been tested on the new SARS-CoV-2 virus, this should not negate considering this approach, especially when effects of probiotics against other coronavirus strains have been reported (42–45). Furthermore, patients are dying from secondary bacterial infections. A recent study in mice has shown that oral administration of *Lactobacillus acidophilus* CMCC878, started 24 h after pulmonary inoculation of *Pseudomonas aeruginosa* and *Staphylococcus aureus* reduced bacterial load in the lungs, and decreased lung damage and systemic inflammation (46).

## Safety of Probiotics

Probiotics are generally safe, even in the most vulnerable populations and in intensive care settings (14, 47). Cases of probiotic-associated bacteremia and fungaemia have occurred on extremely rare occasions, mainly in premature and immunocompromised patients treated with preparations lacking adequate quality control (48, 49). Rather than consider intensive care patients too ill to receive probiotic and prebiotic therapy, RCTs of probiotics for the prevention of ventilator-associated pneumonia provide a reason to consider them (22, 23, 26). Moreover, in an RCT of 65 critically ill, mechanically ventilated, multiple trauma patients, the synbiotic *Pediococcus pentosaceus* 5-33:3, *Leuconostoc mesenteroides* 32-77:1, *L. paracasei* ssp. *paracasei* 19, *L. plantarum* 2,362 plus inulin, oat bran, pectin, and resistant starch resulted in reduced rate of infections, systemic inflammatory response syndrome, sepsis, days of stay in the intensive care unit, days under mechanical ventilation, and mortality (27).

## Summary

In summary, orally administered probiotic strains can reduce the incidence and severity of viral RTIs. At a time when doctors are using drugs with little anti- COVID-19 data, probiotic strains documented for anti-viral and respiratory activities (not low-quality undocumented imitations) should become part of the armamentarium to reduce the burden and severity of this pandemic. Government funding is being used to test numerous drugs but just as important, they should fund probiotic trials. In addition, use of recognized prebiotics (e.g., fructans, galactans) to enhance propagation of probiotic strains and indigenous beneficial microbes should be recommended as part of the overall strategy to flatten the curve (11, 50).

## Author Contributions

EG, DB, and VD contributed conception of the manuscript. EG and VD wrote the first draft. DB, GG, and GR wrote sections of the manuscript. All authors contributed to manuscript revision, read, and approved the submitted version.

## Conflict of Interest

GG and GR provide advice to probiotic and prebiotic companies. The remaining authors declare that the research was conducted in the absence of any commercial or financial relationships that could be construed as a potential conflict of interest.

## References

[1] Ng SC Tilg H. COVID-19 and the gastrointestinal tract: more than meets the eye. Gut. (2020). 10.1136/gutjnl-2020-321195 32273292 PMC7211058

[2] Pan Y Zhang D Yang P Poon LLM Wang Q. Viral load of SARS-CoV-2 in clinical samples. Lancet Infect Dis. (2020) 20:411–2. 10.1016/S1473-3099(20)30113-4 32105638 PMC7128099

[3] Jin X Lian JS Hu JH Gao J Zheng L Zhang YM. Epidemiological, clinical and virological characteristics of 74 cases of coronavirus-infected disease 2019 (COVID-19) with gastrointestinal symptoms. Gut. (2020). [Epub ahead of print]. 10.1136/gutjnl-2020-320926 32213556 PMC7133387

[4] Lin L Jiang X Zhang Z Huang S Zhang Z Fang Z. Gastrointestinal symptoms of 95 cases with SARS-CoV-2 infection. Gut. (2020). [Epub ahead of print]. 10.1136/gutjnl-2020-321013 32241899

[5] Wu F Xiao A Zhang J Gu X Lee WL Kauffman K SARS-CoV-2 titers in wastewater are higher than expected from clinically confirmed cases. medRxiv. (2020). [Epub ahead of print]. 10.1101/2020.04.05.20051540 PMC7566278 32694130

[6] Hill C Guarner F Reid G Gibson GR Merenstein DJ Pot B. Expert consensus document. The International Scientific Association for Probiotics and Prebiotics consensus statement on the scope and appropriate use of the term probiotic. Nat Rev Gastroenterol Hepatol. (2014) 11:506–14. 10.1038/nrgastro.2014.66 24912386

[7] Szajewska H Kolodziej M Gieruszczak-Bialek D Skorka A Ruszczynski M Shamir R. Systematic review with meta-analysis: *Lactobacillus rhamnosus* GG for treating acute gastroenteritis in children–a 2019 update. Aliment Pharmacol Ther. (2019) 49:1376–84. 10.1111/apt.15267 31025399

[8] Goldenberg JZ Yap C Lytvyn L Lo CK Beardsley J Mertz D. Probiotics for the prevention of *Clostridium difficile*-associated diarrhea in adults and children. Cochrane Database Syst Rev. (2017) 12:CD006095. 10.1002/14651858.CD006095.pub4 29257353 PMC6486212

[9] Guo Q Goldenberg JZ Humphrey C El Dib R Johnston BC. Probiotics for the prevention of pediatric antibiotic-associated diarrhea. Cochrane Database Syst Rev. (2019) 4:CD004827. 10.1002/14651858.CD004827.pub5 31039287 PMC6490796

[10] Lenoir-Wijnkoop I Gerlier L Roy D Reid G. The clinical and economic impact of probiotics consumption on respiratory tract infections: projections for Canada. PLoS ONE. (2016) 11:e0166232. 10.1371/journal.pone.0166232 27832195 PMC5104466

[11] Pregliasco F Anselmi G Fonte L Giussani F Schieppati S Soletti L. A new chance of preventing winter diseases by the administration of synbiotic formulations. J Clin Gastroenterol. (2008) 42(Suppl.3):S224–33. 10.1097/MCG.0b013e31817e1c91 18685511

[12] Guillemard E Tondu F Lacoin F Schrezenmeir J. Consumption of a fermented dairy product containing the probiotic *Lactobacillus casei* DN-114001 reduces the duration of respiratory infections in the elderly in a randomised controlled trial. Br J Nutr. (2010) 103:58–68. 10.1017/S0007114509991395 19747410

[13] Guillemard E Tanguy J Flavigny A de la Motte S Schrezenmeir J. Effects of consumption of a fermented dairy product containing the probiotic *Lactobacillus casei* DN-114 001 on common respiratory and gastrointestinal infections in shift workers in a randomized controlled trial. J Am Coll Nutr. (2010) 29:455–68. 10.1080/07315724.2010.10719882 21504972

[14] Dermyshi E Wang Y Yan C Hong W Qiu G Gong X. The “golden age” of probiotics: a systematic review and meta-analysis of randomized and observational studies in preterm infants. Neonatology. (2017) 112:9–23. 10.1159/000454668 28196365

[15] Panigrahi P Parida S Nanda NC Satpathy R Pradhan L Chandel DS. A randomized synbiotic trial to prevent sepsis among infants in rural India. Nature. (2017) 548:407–12. 10.1038/nature23480 28813414

[16] de Vrese M Winkler P Rautenberg P Harder T Noah C Laue C. Effect of *Lactobacillus gasseri* PA 16/8, *Bifidobacterium longum* SP 07/3, *B. bifidum* MF 20/5 on common cold episodes: a double blind, randomized, controlled trial. Clin Nutr. (2005) 24:481–91. 10.1016/j.clnu.2005.02.006 16054520

[17] Luoto R Ruuskanen O Waris M Kalliomaki M Salminen S Isolauri E. Prebiotic and probiotic supplementation prevents rhinovirus infections in preterm infants: a randomized, placebo-controlled trial. J Allergy Clin Immunol. (2014) 133:405–13. 10.1016/j.jaci.2013.08.020 24131826 PMC7112326

[18] Waki N Matsumoto M Fukui Y Suganuma H. Effects of probiotic *Lactobacillus brevis* KB290 on incidence of influenza infection among schoolchildren: an open-label pilot study. Lett Appl Microbiol. (2014) 59:565–71. 10.1111/lam.12340 25294223 PMC4285317

[19] Namba K Hatano M Yaeshima T Takase M Suzuki K. Effects of *Bifidobacterium longum* BB536 administration on influenza infection, influenza vaccine antibody titer, and cell-mediated immunity in the elderly. Biosci Biotechnol Biochem. (2010) 74:939–45. 10.1271/bbb.90749 20460726

[20] Rautava S Salminen S Isolauri E. Specific probiotics in reducing the risk of acute infections in infancy–a randomised, double-blind, placebo-controlled study. Br J Nutr. (2009) 101:1722–6. 10.1017/S0007114508116282 18986600

[21] Cardenas N Martin V Arroyo R López M Carrera M Badiola C. Prevention of recurrent acute otitis media in children through the use of *Lactobacillus salivarius* PS7, a target-specific probiotic strain. Nutrients. (2019) 11:e376. 10.3390/nu11020376 30759799 PMC6413216

[22] Bo L Li J Tao T Bai Y Ye X Hotchkiss RS Probiotics for preventing ventilator-associated pneumonia. Cochrane Database Syst Rev. (2014) 10:CD009066 10.1002/14651858.CD009066.pub2 PMC4283465 25344083

[23] Su M Jia Y Li Y Zhou D Jia J. Probiotics for the prevention of ventilator-associated pneumonia: a meta-analysis of randomized controlled trials. Respir Care. (2020) 65:5. [Epub ahead of print]. 10.4187/respcare.07097 32127415

[24] Lehtoranta L Kalima K He L Lappalainen M Roivainen M Närkiö M. Specific probiotics and virological findings in symptomatic conscripts attending military service in Finland. J Clin Virol. (2014) 60:276–81. 10.1016/j.jcv.2014.03.021 24793963 PMC7185430

[25] Chong HX Yusoff NAA Hor YY Lew LC Jaafar MH Choi SB. *Lactobacillus plantarum* DR7 improved upper respiratory tract infections via enhancing immune and inflammatory parameters: a randomized, double-blind, placebo-controlled study. J Dairy Sci. (2019) 102:4783–97. 10.3168/jds.2018-16103 30954261

[26] Shimizu K Yamada T Ogura H Mohri T Kiguchi T Fujimi S. Synbiotics modulate gut microbiota and reduce enteritis and ventilator-associated pneumonia in patients with sepsis: a randomized controlled trial. Crit Care. (2018) 22:239. 10.1186/s13054-018-2167-x 30261905 PMC6161427

[27] Kotzampassi K Giamarellos-Bourboulis EJ Voudouris A Kazamias P Eleftheriadis E. Benefits of a synbiotic formula (Synbiotic 2000Forte) in critically Ill trauma patients: early results of a randomized controlled trial. World J Surg. (2006) 30:1848–55. 10.1007/s00268-005-0653-1 16983476

[28] Bermudez-Brito M Plaza-Diaz J Munoz-Quezada S Gomez-Llorente C Gil A. Probiotic mechanisms of action. Ann Nutr Metab. (2012) 61:160–74. 10.1159/000342079 23037511

[29] Saavedra JM Bauman NA Oung I Perman JA Yolken RH. Feeding of *Bifidobacterium bifidum* and *Streptococcus thermophilus* to infants in hospital for prevention of diarrhoea and shedding of rotavirus. Lancet. (1994) 344:1046–9. 10.1016/S0140-6736(94)91708-6 7934445

[30] Gonzalez-Ochoa G Flores-Mendoza LK Icedo-Garcia R Gomez-Flores R Tamez-Guerra P. Modulation of rotavirus severe gastroenteritis by the combination of probiotics and prebiotics. Arch Microbiol. (2017) 199:953–61. 10.1007/s00203-017-1400-3 28634691 PMC5548957

[31] Enaud R Prevel R Ciarlo E Beaufils F Wieërs G Guery B. The gut-lung axis in health and respiratory diseases: a place for inter-organ and inter-kingdom crosstalks. Front Cell Infect Microbiol. (2020) 10:9. 10.3389/fcimb.2020.00009 32140452 PMC7042389

[32] Sencio V Barthelemy A Tavares LP Machado MG Soulard D Cuinat C. Gut dysbiosis during influenza contributes to pulmonary pneumococcal superinfection through altered short-chain fatty acid production. Cell Rep. (2020) 30:2934–47 e6. 10.1016/j.celrep.2020.02.013 32130898

[33] Hanada S Pirzadeh M Carver KY Deng JC. Respiratory viral infection-induced microbiome alterations and secondary bacterial pneumonia. Front Immunol. (2018) 9:2640. 10.3389/fimmu.2018.02640 30505304 PMC6250824

[34] Gao QY Chen YX Fang JY 2019 Novel coronavirus infection and gastrointestinal tract. J Dig Dis. (2020) 12:3 10.1111/1751-2980.12851 PMC7162053 32096611

[35] Xu K Cai H Shen Y Ni Q Chen Y Hu S. Management of corona virus disease-19 (COVID-19): the Zhejiang experience. Zhejiang Da Xue Xue Bao Yi Xue Ban. (2020) 49. 32391658 10.3785/j.issn.1008-9292.2020.02.02 PMC8800711

[36] Di Pierro F. A possible probiotic (*S. salivarius* K12) approach to improve oral and lung microbiotas and raise defenses against SARS-CoV-2. Minerva Med. (2020). [Epub ahead of print]. 10.23736/S0026-4806.20.06570-2 32255312

[37] Arroyo R Martin V Maldonado A Jimenez E Fernandez L Rodriguez JM. Treatment of infectious mastitis during lactation: antibiotics versus oral administration of lactobacilli isolated from breast milk. Clin Infect Dis. (2010) 50:1551–8. 10.1086/652763 20455694

[38] Abt MC Osborne LC Monticelli LA Doering TA Alenghat T Sonnenberg GF. Commensal bacteria calibrate the activation threshold of innate antiviral immunity. Immunity. (2012) 37:158–70. 10.1016/j.immuni.2012.04.011 22705104 PMC3679670

[39] Zelaya H Alvarez S Kitazawa H Villena J. Respiratory antiviral immunity and immunobiotics: beneficial effects on inflammation-coagulation interaction during influenza virus infection. Front Immunol. (2016) 7:633. 10.3389/fimmu.2016.00633 28066442 PMC5179578

[40] Ichinohe T Pang IK Kumamoto Y Peaper DR Ho JH Murray TS. Microbiota regulates immune defense against respiratory tract influenza A virus infection. Proc Natl Acad Sci USA. (2011) 108:5354–9. 10.1073/pnas.1019378108 21402903 PMC3069176

[41] Turner RB Woodfolk JA Borish L Steinke JW Patrie JT Muehling LM. Effect of probiotic on innate inflammatory response and viral shedding in experimental rhinovirus infection–a randomised controlled trial. Benef Microbes. (2017) 8:207–15. 10.3920/BM2016.0160 28343401 PMC5797652

[42] Kumar R Seo BJ Mun MR Kim CJ Lee I Kim H. Putative probiotic *Lactobacillus* spp. from porcine gastrointestinal tract inhibit transmissible gastroenteritis coronavirus and enteric bacterial pathogens. Trop Anim Health Prod. (2010) 42:1855–60. 10.1007/s11250-010-9648-5 20623187 PMC7089342

[43] Chai W Burwinkel M Wang Z Palissa C Esch B Twardziok S. Antiviral effects of a probiotic *Enterococcus faecium* strain against transmissible gastroenteritis coronavirus. Arch Virol. (2013) 158:799–807. 10.1007/s00705-012-1543-0 23188495 PMC7086644

[44] Liu YS Liu Q Jiang YL Yang WT Huang HB Shi CW. Surface-displayed porcine IFN-lambda3 in *Lactobacillus plantarum* inhibits porcine enteric coronavirus infection of porcine intestinal epithelial cells. J Microbiol Biotechnol. (2019) 30:515–525. [Epub ahead of print]. 10.4014/jmb.1909.09041 31838830 PMC9728374

[45] Wang K Ran L Yan T Niu Z Kan Z Zhang Y. Anti-TGEV miller strain infection effect of *Lactobacillus plantarum* supernatant based on the JAK-STAT1 signaling pathway. Front Microbiol. (2019) 10:2540. 10.3389/fmicb.2019.02540 31781061 PMC6851170

[46] Shoaib A Xin L Xin Y. Oral administration of *Lactobacillus acidophilus* alleviates exacerbations in *Pseudomonas aeruginosa* and *Staphylococcus aureus* pulmonary infections. Pak J Pharm Sci. (2019) 32:1621–30. 31608882

[47] Sanders ME Merenstein DJ Ouwehand AC Reid G Salminen S Cabana MD. Probiotic use in at-risk populations. J Am Pharm Assoc. (2016) 56:680–6. 10.1016/j.japh.2016.07.001 27836128

[48] Bertelli C Pillonel T Torregrossa A Simonsen GS Støen R Klingenberg C. *Bifidobacterium longum* bacteremia in preterm infants receiving probiotics. Clin Infect Dis. (2015) 60:924–7. 10.1093/cid/ciu946 25472946

[49] Kolacek S Hojsak I Berni Canani R Guarino A Indrio F Orel R. Commercial probiotic products: a call for improved quality control. A position paper by the ESPGHAN Working Group for Probiotics and Prebiotics. J Pediatric Gastroenterol Nutr. (2017) 65:117–24. 10.1097/MPG.0000000000001603 28644359

[50] Chan CKY Tao J Chan OS Li HB Pang H. Preventing respiratory tract infections by synbiotic interventions: a systematic review and meta-analysis of randomized controlled trials. Adv Nutr. (2020). [Epub ahead of print]. 10.1093/advances/nmaa003 31996911 PMC7360463

